# Evaluation of rational nonsteroidal anti-inflammatory drugs and gastro-protective agents use; association rule data mining using outpatient prescription patterns

**DOI:** 10.1186/s12911-017-0496-3

**Published:** 2017-07-04

**Authors:** Oraluck Pattanaprateep, Mark McEvoy, John Attia, Ammarin Thakkinstian

**Affiliations:** 1 Section for Clinical Epidemiology and Biostatistics, The Faculty of Medicine Ramathibodi Hospital, Mahidol University, 270 Rama VI Rd., Ratchathewi, Bangkok, 10400 Thailand; 2 Centre for Clinical Epidemiology and Biostatistics, and Hunter Medical Research Institute, The University of Newcastle, Newcastle, NSW Australia

**Keywords:** Data mining, Association rule, Apriori algorithm, Prescription patterns, Rational drug use, Hospital, Data warehouse, Nonsteroidal anti-inflammatory drugs, Gastro-protective agents

## Abstract

**Background:**

Nonsteroidal anti-inflammatory drugs (NSAIDs) and gastro-protective agents should be co-prescribed following a standard clinical practice guideline; however, adherence to this guideline in routine practice is unknown. This study applied an association rule model (ARM) to estimate rational NSAIDs and gastro-protective agents use in an outpatient prescriptions dataset.

**Methods:**

A database of hospital outpatients from October 1st, 2013 to September 30th, 2015 was searched for any of following drugs: oral antacids (A02A), peptic ulcer and gastro-oesophageal reflux disease drugs (GORD, A02B), and anti-inflammatory and anti-rheumatic products, non-steroids or NSAIDs (M01A). Data including patient demographics, diagnoses, and drug utilization were also retrieved. An association rule model was used to analyze co-prescription of the same drug class (i.e., prescriptions within A02A-A02B, M01A) and between drug classes (A02A-A02B & M01A) using the Apriori algorithm in R. The lift value, was calculated by a ratio of confidence to expected confidence, which gave information about the association between drugs in the prescription.

**Results:**

We identified a total of 404,273 patients with 2,575,331 outpatient visits in 2 fiscal years. Mean age was 48 years and 34% were male. Among A02A, A02B and M01A drug classes, 12 rules of associations were discovered with support and confidence thresholds of 1% and 50%. The highest lift was between Omeprazole and Ranitidine (340 visits); about one-third of these visits (118) were prescriptions to non-GORD patients, contrary to guidelines. Another finding was the concomitant use of COX-2 inhibitors (Etoricoxib or Celecoxib) and PPIs. 35.6% of these were for patients aged less than 60 years with no GI complication and no Aspirin, inconsistent with guidelines.

**Conclusions:**

Around one-third of occasions where these medications were co-prescribed were inconsistent with guidelines. With the rapid growth of health datasets, data mining methods may help assess quality of care and concordance with guidelines and best evidence.

## Background

Nonsteroidal anti-inflammatory drugs (NSAIDs) are used to relieve pain and inflammation. However, conventional NSAIDs (e.g., Diclofenac, Meloxicam, Ibuprofen) can induce gastrointestinal (GI) upset and adverse events, especially peptic ulceration [1]. To reduce this risk, gastro-protective agents are commonly co-prescribed with NSAIDs; alternatively, cyclooxygenase (COX)-2 inhibitors (e.g., Etoricoxib, Celecoxib) are used, a new generation of NSAIDs claimed to cause fewer gastrointestinal adverse events [2–4]. Co-prescription of COX-2 inhibitors with gastro-protective agents are recommended only in patients at high risk of GI disease, such as elderly patients (aged ≥ 60 years), those using antiplatelet agents (e.g., Aspirin), or patients with a history of GI events [2, 5].

Commonly used gastro-protective agents are histamine H2-receptor antagonists (H2RAs, e.g., Ranitidine) and proton pump inhibitors (PPIs, e.g., Omeprazole, Pantoprazole, Esomeprazole, Lansoprazole). The H2RAs competitively antagonize the histamine effects at H2-receptors in the stomach to reduce the amount and concentration of gastric acid. PPIs suppress stomach acid secretion by specific inhibition of the H^+^/K^±^ ATPase system found at the secretory surface of gastric parietal cells [6–9]. Concomitant use of H2RAs and PPIs are recommended only in the treatment of gastro-oesophageal reflux disease (GORD) [10, 11].

In the past, identification of poor quality drug use in the hospital was not easily done, because of the volume and complexity of prescription data. In our institution (Ramathibodi Hospital, Bangkok, Thailand) data warehouses have been available since 2014, and there has been interest in using these to drive quality improvement in health care practice and service delivery. These data include drug prescriptions, demographic data, diagnoses, laboratory tests, imaging, etc., and are routinely extracted from hospital information systems (HIS).

Currently, a wide variety of data mining algorithms (i.e., technique for big data analysis) are available; they are classified into 2 main categories: supervised and unsupervised learning [12]. Supervised learning algorithms produce a model using classification or regression that can predict the response values for a particular outcome or behavior of interest. Unsupervised learning algorithms describe the form and hidden structure of data, using methods such as clustering, anomaly detection, and association rule mining (ARM), which has been applied for detecting co-prescription patterns in many studies [13–17].

The Apriori algorithm is a classical ARM technique, based on the principle of frequent pattern mining [18–21]. First, a candidate set is generated to identify items that occur with a frequency that exceeds a pre-specified threshold (i.e., defined as the support measure). Second, the association rules are derived by indicating conditional probabilities between a pair of items; groups are defined if the conditional probability value exceeds a user-defined threshold (called the confidence measure).

Our study aimed to assess associations within the gastro-protective agents (H2RAs and PPIs), and NSAIDs (including COX-2 inhibitors), as well as between these two drug classes using ARM. Once associations were identified, prescription patterns were explored for congruence with guidelines.

## Methods

An electronic database of outpatients records at Ramathibodi Hospital between October 1st, 2013 and September 30th, 2015 was extracted from the hospital data warehouse focusing on H2RAs and PPIs (A02A and A02B codes), and NSAIDs and COX-2 inhibitors (M01A). Only fields for patient demographics, prescriptions, drug utilization, and diagnoses were retrieved. Two steps of data manipulation and analysis were then performed using R software version 3.3.0 in RStudio® version 0.99.902 (RStudio Inc., Boston, MA, USA). First, the data frame was constructed and then data was analyzed to identify association rules and evaluate rational drug use.

### Data retrieval and manipulation

Five tables in the hospital data warehouse were retrieved as follows: 1) physician prescriptions, 2) master drug lists, 3) drug utilization, 4) diagnosis data, and 5) patient demographic data. The study protocol was approved by the ethics committee of Ramathibodi Hospital without requirement of consent for participation. As for our hospital’s rule, data were not available for public and thus we could not provide and share individual patient data.

The physician prescriptions over 2 fiscal years were retrieved. These data had been already cleaned through an “Extract, Transform, Load” (ETL) process while being loaded into the data warehouse on a daily basis [22]. Master drug lists from the data warehouse were also loaded and merged in RStudio®. To manipulate the data frame, R commands were constructed and run to select ambulatory or outpatient prescriptions with Anatomical Therapeutic Chemical (ATC) classification system codes of A02A: Antacids, A02B: Drug for peptic ulcer and GORD, and M01A: Anti-inflammatory and anti-rheumatic products, non-steroids or NSAIDs (see Table 1).

**Table 1:** Drug code of 1A and 4 L drugs and their names

Drug code	Name	Drug code	Name
A02A - Antacids		M01AA to M01AG, M01AX – conventional NSAIDs	
ALGY-T-	Alginic acid tablet	ASPT-T-	Acetylsalicylic acid 300 mg
ALHY-T-	Aluminium hydroxide 500 mg	ASA.1 T-	Acetylsalicylic acid 81 mg
ALHY-N2	Aluminium hydroxide 6.10%	CAPN-T-	Acetylsalicylic acid 100 mg
ANTT-T-	Aluminium hydroxide tablet	CLIR-T-	Sulindac 200 mg
ANTC-N2	Aluminium hydroxide 1000 ml	DICF-T-	Diclofenac 25 mg
ANTC-N1	Aluminium hydroxide 240 ml	FAFX-T-	Nabumetone 500 mg
GAVD-T-	Sodium alginate Dual Action	FLAM-C-	Piroxicam 10 mg
GAVI-N-	Sodium alginate Liquid	IBUP-S-	Ibuprofen (100 mg/5 ml)
GAST-T-	Bismuth subsalicylate 524 mg	IBUP1T-	Ibuprofen 200 mg
MUCT-T-	Rebamipide 100 mg	IBUP2T-	Ibuprofen 400 mg
ULCF-N-	Sucralfate 240 ml	INDM-C-	Indomethacin 25 mg
ULCF-N1	Sucralfate 60 ml	MEFN1C-	Mefenamic acid 250 mg
ULSN-T-	Sucralfate 500 mg	MEFN2T-	Mefenamic acid 500 mg
ULSN1T-	Sucralfate 1000 mg	MELO-T-	Meloxicam 7.5 mg
A02B – Drug for peptic ulcer and GORD		MELO1T-	Meloxicam 15 mg
CYTT-T-	Misoprostol 200 mcg	NAPS-T-	Naproxen LE 250 mg
XAND-T-	Ranitidine 150 mg	NAPX-T-	Naproxen 250 mg
COTL2T-	Pantoprazole 20 mg	REMT-T-	Diclofenac 100 mg
COTL-T-	Pantoprazole 40 mg	VOLS1T-	Diclofenac SR 75 mg
DEXI1C-	Dexlansoprazole 60 mg	VOLS-T-	Diclofenac SR 100 mg
DEXI-C-	Dexlansoprazole 30 mg	M01AH – COX-2 inhibitors	
LOSC-C-	Omeprazole MUPS 20 mg	ARCX4T-	Etoricoxib 30 mg
NEXM1T-	Esomeprazole 20 mg	ARCX1T-	Etoricoxib 60 mg
NEXM2T-	Esomeprazole 40 mg	ARCX2T-	Etoricoxib 90 mg
OMPZ-C-	Omeprazole 20 mg	CELB-C-	Celecoxib 200 mg
PARI-T-	Rabeprazole 10 mg	CELB1C-	Celecoxib 400 mg
PARI1T-	Rabeprazole 20 mg		
PRVF1T-	Lansoprazole 15 mg		
PRVF2T-	Lansoprazole 30 mg		

*Note*: every patient in the cohort was prescribed at least one of the listed drugs

Two years of data were combined and drug strength and dosage were ascertained from the left 4 digits of the drug code substring, e.g. IBUP1T- (Ibuprofen 200 mg tablet), IBUP2T- (Ibuprofen 400 mg tablet), IBUP-S- (Ibuprofen 100 mg/5 ml) syrup transformed to the same code - IBUP for Ibuprofen. HN (patient’s hospital number) and date were joined to create HNDate, to represent visit date. Data frame was reshaped from long to wide format e.g.
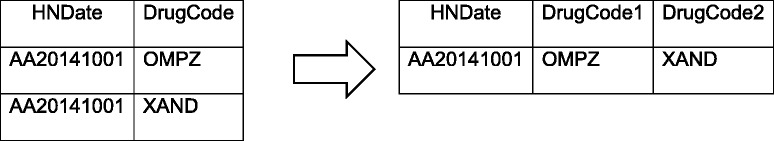


And records with only one drug item per patient per day were excluded.

Drug utilization, diagnosis data, and patients’ demographic data were also retrieved from tables in the hospital data warehouse to get each prescription’s dose and frequency, primary/secondary diagnosis of each visit (with International Classification of Disease, Tenth Edition ICD-10), date of birth (to calculate age), and gender. All data were merged with physician prescriptions by HNDate.

### Data analysis

Patient age and number of OPD visits/person/year were described using mean (SD) and number of male and number of diagnoses, defined by ICD-10 codes: K20-K29.9, K30-K38.9, K90-K93.8 for gastrointestinal complications. The Apriori algorithm with ARM was applied to assess the pattern of associations within the same drug classes (i.e., gastro-protective agents, NSAIDs) and between different drug classes (i.e., gastro-protective agents and NSAIDs).

Association rules were derived based on prescription data. The rules were aimed to detect prescribing patterns of NSAIDs and gastro-protective agents for individual patients in the same visit with detail as follows: Let *I* be a set of prescribed drug items (i.e., NSAIDs and gastro-protective agents) listed in the database and *P* = {*P*_1_, *P*_2_,…, *P*_n_} be a set of number of prescriptions, where *P*_i_ (1 ≤ i ≤ n) is a set of drugs in prescription i. Given X and Y as non-overlapping sets of drug items (i.e., X ∩ Y = ∅), the ARM is used to measure how often X (called antecedent or left-hand-side or LHS) and Y (called consequent or right-hand-side or RHS) occurred/appeared together in the same prescription (*P*_i_). The association rules use 3 probability estimations: support, confidence, and lift without adjusting for derivation of multiple sets of drug items. Support is defined as the probability of prescriptions in *P* contains X and Y, i.e., support(X➔Y) = P(X∪Y). Confidence is defined as the conditional probability of having Y given X; confidence(X➔Y) = P(Y|X). Lift is the deviation of the support parameter from what would be expected if X and Y were independent; lift(X➔Y) = P(X,Y) / P(X) x P(Y); lift values of <1, >1, and 1 refer to negative, positive, and independent associations between X and Y, respectively [20, 21, 23].

The Apriori algorithm in R was used for analyzing the ARM parameters with the command [24] as

$$ \mathrm{Apriori}\ \left(\mathrm{data},\mathrm{parameter}=\mathrm{NULL},\mathrm{appearance}=\mathrm{NULL},\mathrm{control}=\mathrm{NULL}\right) $$

From ARM, related data in 3 tables including drug utilization, diagnosis data, and patients’ demographic data, were explored and assessed to evaluate rational use of 2 concomitant drugs. In the first group - concomitant use of H2RAs and PPIs - dose and frequency appearing in each prescription along with clinic data were cross-checked for drug interaction or over-dosage. Number and percentage of prescriptions for any concomitant use of H2RAs and PPIs were compared with GORD (described in primary/secondary diagnosis).

In the second group - concomitant use of COX-2 inhibitors and PPIs - patients’ characteristics, number and percentage of prescriptions by age groups, co-therapy with Aspirin, and GI complication were described.

## Results

A total of 2,575,331 outpatient visits over 2 fiscal years were retrieved. The mean age and number of OPD visits were 48.4 (SD = 21.4) years and 4.7 (SD = 4.4) per person per year, respectively, and the majority were females (66%). The percentages with GI complications and arthritis were 1.80% and 0.74%, respectively. Among them, 134,285 prescriptions had at least one oral antacid (A02A), drug for peptic ulcer and GORD (A02B), or NSAIDs (M01A) in the same day. A total of 128,117 (95.4%) observations were omitted due to prescription of only one drug per visit, leaving 6168 observations for ARM analysis.

The ARM was applied starting with a threshold of 1% for both support and confidence parameters, and increasing the threshold until association rules were found. Twelve rules were identified and pass the thresholds of 1% and 50% for support and confidence parameters, respectively (see Table 2). The strongest support parameter (0.2244) was between Aspirin and Omeprazole. The strongest confidence parameter (0.9738) was between Naproxen and Omeprazole. Lift values of <1, >1, and 1 refer to negative, positive, and independent associations between antecedent and consequent, respectively, the larger of the value indicates the more significant of the association. The most significant association was between Omeprazole and Ranitidine with highest lift of 7.6153. The rest was low associations between other drugs and Omeprazole.

**Table 2:** LHS, RHS, support, confidence and lift of 12 rules

Rule no.	LHS	RHS	Support	Confidence	Lift
1	OMPZ	XAND	0.0552	0.7944	7.6153
2	XAND	OMPZ	0.0552	0.5288	7.6153
3	NAPX	OMPZ	0.1085	0.9738	1.4363
4	MELO	OMPZ	0.0315	0.9557	1.4096
5	IBUP	OMPZ	0.0362	0.9028	1.3317
6	DICF	OMPZ	0.0109	0.8933	1.3177
7	ASPT	OMPZ	0.0399	0.8723	1.2867
8	ASA.	OMPZ	0.2244	0.7840	1.1564
9	CELB	OMPZ	0.0483	0.7582	1.1184
10	MOBC	OMPZ	0.0133	0.7522	1.1096
11	ARCX	OMPZ	0.0860	0.6901	1.0179
12	ANTC	OMPZ	0.0315	0.5543	0.8176

Among these 12 association rules, the number of prescriptions of concomitant use for the first and second lifts (i.e., H2RAs and PPIs and COX-2 inhibitors and PPIs) were next calculated. For H2RAs and PPIs (i.e., Ranitidine and Omeprazole), the support and numbers of observations were 0.0552 and 6168, respectively. As a result, 340 (0.0552 × 6168) visits were prescribed with Omeprazole and Ranitidine on the same day.

Since Omeprazole and Ranitidine are in the same drug class (A02B) for treatment of GORD, rational concomitant drug uses for these 340 visits were therefore explored, see Table 3. Drug dose and frequency from each prescription were retrieved. Among these, one patient was prescribed both drugs from different clinics, 12 patients were prescribed Omeprazole and Ranitidine by the same physicians with taking both drugs at the same meals, while the rest of the patients received two drugs from one physician but for different meals. All GI related diagnoses were further explored among these 340 patients, see Table 4. The results indicate that in 118 visits or one-third of these patients, the combination was not prescribed for GORD.

**Table 3:** Drug’s dose and frequency of Omeprazole (OMPZ) and Ranitidine (XAND)

Code	Dose and frequency			Clinic	Code	Dose and frequency			Clinic
OMPZ	1	CAP	AM	SDORP11	XAND	1	TAB	BID	OFM18
OMPZ	1	CAP	AM	SDPMD02	XAND	1	TAB	BID	SDPMD02
OMPZ	1	CAP	AM	OGY111	XAND	1	TAB	BID	OGY111
OMPZ	2	CAP	AM	OPS01	XAND	1	TAB	BID	OPS01
OMPZ	1	CAP	AM	SDOET11	XAND	1	TAB	BID	SDOET11
OMPZ	1	CAP	AM	SDPMD02	XAND	1	TAB	BID	SDPET01
OMPZ	1	CAP	BID	OPS02	XAND	1	TAB	BID	OPS02
OMPZ	1	CAP	BID	SDPET01	XAND	1	TAB	BID	SDPET01
OMPZ	1	CAP	BID	OEX01	XAND	1	TAB	BID	OEX01
OMPZ	1	CAP	BID	SDPET01	XAND	1	TAB	BID	SDPET01
OMPZ	1	CAP	BID	SDOSU05	XAND	1	TAB	BID	SDOSU05
OMPZ	1	CAP	BID	SDPRP03	XAND	2	TAB	PM	SDPRP03
OMPZ	2	CAP	BID	SDPRP03	XAND	1	TAB	PM	SDPRP03

*CAP* capsule, *TAB* tablet, *AM* in a morning, *PM* in an evening, *BID* twice a day, in morning and evening

**Table 4:** Diagnosis related to GI complications of visits prescribed Omeprazole and Ranitidine on the same day, frequency (%)

ICD10	Disease	*N* = 340
K219	Gastro-oesophageal reflux disease without oesophagitis	221 (65.0)
K259	Gastric ulcer Unspecified as acute or chronic, without haemorrhage or perforation	1 (0.3)
	GORD	222
K30	Dyspepsia	38 (11.2)
K279	Peptic ulcer, site unspecified Unspecified as acute or chronic, without haemorrhage or perforation	9 (2.6)
K297	Gastritis, unspecified	5 (1.5)
K922	Gastrointestinal haemorrhage, unspecified	2 (0.6)
K921	Melaena	1 (0.3)
K319	Disease of stomach and duodenum, unspecified	1 (0.3)
K254	Gastric ulcer Chronic or unspecified with haemorrhage	1 (0.3)
K210	Gastro-oesophageal reflux disease with oesophagitis	1 (0.3)
K20	Oesophagitis	1 (0.3)
	Non-GORD	118

In the second group, we looked at concomitant use of COX-2 inhibitors with PPIs, a combination that is indicated only in elderly patients or those who have GI complications or are taking Aspirin. From a total of 828 visits, there were no COX-2 inhibitors (i.e., Etoricoxib or Celecoxib) prescribed in the same visit. Of these, 295 (35.6%) visits (Table 5) did not comply with the clinical practice guidelines, i.e. for patients aged less than 60 years with no GI complication and no Aspirin taken.

**Table 5:** Category of visits prescribed COX-2 inhibitors (Etoricoxib or Celecoxib) with Omeprazole, frequency (%)

Category		*N* = 828
Age (years)	> = 60	498 (60.2)
	50–59	233 (28.1)
	< 40	97 (11.7)
GI complication (K20-K29.9, K30-K38.9, K90-K93.8)	Yes	141 (17.0)
	No	687 (83.0)
Aspirin taken	Yes	11 (1.3)
	No	917 (98.7)
Age < 60 years with no GI complication and no Aspirin taken	Yes	295 (35.6)
	No	533 (64.4)

## Discussion

The study applied ARM to find association rules in prescribing drugs that contained any of 2 drug groups in the same day, i.e., NSAIDs and gastro-protective agents. Data were manipulated and analyzed by Apriori algorithm in RStudio®. Twelve rules were found with >1% support and >50% confidence thresholds and revealed 2 non-guideline prescription patterns of NSAIDs and gastro-protective agents from a hospital data warehouse i.e., Omeprazole with Ranitidine, and COX-2 inhibitors with Omeprazole.

The overwhelming majority of prescriptions (95%) were only for single agents, indicating that rational drug prescriptions was occurring the majority of the time. However, the remaining 5% still represented over 6000 prescriptions and these need more analysis to ascertain whether they complied with clinical practice guidelines.

Among scripts with more than one drug, the strongest association was between Omeprazole and Ranitidine, both of which are in the same drug group, (A02B). Although their pharmacological pathways are different [5], most physicians prescribe either one or another. However, evidence from few studies indicated that taking these 2 drugs in the same meal can improve gastric acid control [10, 11].

The second prescription pattern was between COX-2 inhibitors and Omeprazole. There is no cost effectiveness study directly supporting the benefits of this combination strategy [25], and PPIs are clinically not indicated to prescribe with COX-2 inhibitors, except for high GI risk patients [5].

This study showed that ARM could detect possible poor quality of drug prescription patterns from a hospital data warehouse. Applying this ARM in a routine practice of drug prescriptions should support and lead to health care improvement. The ARM has also found benefits in other clinical studies to identify risk patterns for type 2 diabetes [26], analyze the records of patients diagnosed with essential hypertension [27], identify interesting patterns of infection control [28], find disease association rules from the national health insurance research database in Taiwan [29], and to identify product–multiple adverse event associations in the US Vaccine Adverse Event Reporting System (VAERS) [30]. Apriori is an algorithm for generating association rules; other ARM algorithms are Eclat and FP-Growth algorithms [31, 32].

## Conclusion

This study used data in a hospital data warehouse to explore the prescription pattern of 2 drug groups. The method uses an existing algorithm (Apriori) within an open source package (R) for deriving the association rules. Twelve rules were found, representing around one-third of visits (i.e., 118 of 340 who were prescribed Omeprazole with Ranitidine and 295 from 828 who were prescribed Omeprazole with Etoricoxib or Celecoxib), where prescriptions were potentially not congruent with guidelines. This Apriori algorithm should be implemented in hospital monitoring systems in order to detect guideline-discordant use of medicines and routinely feedback to prescribers for increased patient safety.
